# Comparing performance of seven fine-tuned open-source large language models in summarizing and predicting outcome-relevant information from mechanical thrombectomy reports in patients with acute ischemic stroke

**DOI:** 10.1007/s00330-025-12122-x

**Published:** 2025-11-17

**Authors:** Semil Eminovic, Maximilian Lindholz, Andrea Dell’Orco, Aymen Meddeb, Georg Lukas Baumgärtner, Sophia Schulze-Weddige, Eberhard Siebert, Mike P. Wattjes, Tobias Penzkofer, Jawed Nawabi

**Affiliations:** 1 Department of Radiology, Charité – Universitätsmedizin Berlin, Humboldt-Universität zu Berlin, Freie Universität Berlin, Berlin Institute of Health, Berlin, Germany; 2 Berlin Institute of Health at Charité – Universitätsmedizin Berlin, Berlin, Germany; 3 Department of Neuroradiology, Charité – Universitätsmedizin Berlin, Humboldt-Universität zu Berlin, Freie Universität Berlin, Berlin Institute of Health, Berlin, Germany

**Keywords:** Large language models, Stroke, Thrombectomy

## Abstract

**Purpose:**

This study evaluates seven open-source Large Language Models (LLMs) in summarizing radiology reports of acute ischemic stroke patients treated with mechanical thrombectomy and predicting angiography-based outcome measures relevant to post-thrombectomy reperfusion.

**Materials and methods:**

2000 mechanical thrombectomy reports (findings and summarizing impression section as gold standard) were split into training set (*N* = 1900) for model fine-tuning and test set (*N* = 100). A two-step evaluation was performed: (1) Quantitative analyses of seven LLMs with metrics ROUGE-1, -2, -L, METEOR, BERTScore (F1) and BLEU comparing LLM-generated summaries against gold-standard impressions. (2) Qualitative manual evaluation of the four best-performing models by two radiologists, assessing correctness and completeness across key parameters: outcome-relevant scores, vessel information, occlusion side, number of passes, relevant additional information, hallucinations, and grammar quality. Statistical significance was assessed via a two-tailed, four-sample χ² test, followed by post hoc pairwise χ² comparisons.

**Results:**

BioMistral-7b scored highest across most quantitative metrics (ROUGE-1: 0.47, ROUGE-2: 0.30, ROUGE-L: 0.43, METEOR: 0.46, BERTScore (F1): 0.82). Manual evaluation revealed gemma-2-9b most frequently documented pass counts (56 out of 100 cases (56%); *p* < 0.02 vs. Llama-3.1-8b/mistral-7b-instruct), while mistral-7b-instruct described them most often correctly (29 out of 38 mentioned passes (76.32%); *p* < 0.02 vs. BioMistral-7b and *p* < 0.01 vs. gemma-2-9b). All four manually evaluated LLMs performed moderately well in predicting “Thrombolysis-In-Cerebral-Ischemia (TICI)” Score (correctness rate ranging from 66 to 71%; *p* = 0.89).

**Conclusion:**

All four manually evaluated LLMs effectively summarized thrombectomy reports and demonstrated moderate accuracy predicting TICI scores. Their integration into radiology workflows could enhance efficiency, warranting further clinical validation.

**Key Points:**

***Question*** *Specifically fine-tuned Large Language Models (LLMs) can improve radiology workflow by automatically summarizing thrombectomy reports and inferring angiographic classifications from textual descriptions.*

***Findings*** *Fine-tuned LLMs achieve similar performance in summarizing thrombectomy reports, with each model performing best in specific categories and showing moderate accuracy in correct “Thrombolysis-In-Cerebral-Ischemia (TICI)” Score prediction (66–71%).*

***Clinical relevance*** *Integrating fine-tuned LLMs into radiology workflows may accelerate decision-making and improve patient outcomes by automatically summarizing reports and assessing recanalization success, while future work should enhance contextual understanding, address ambiguous inputs, and limit hallucinations.*

**Graphical Abstract:**

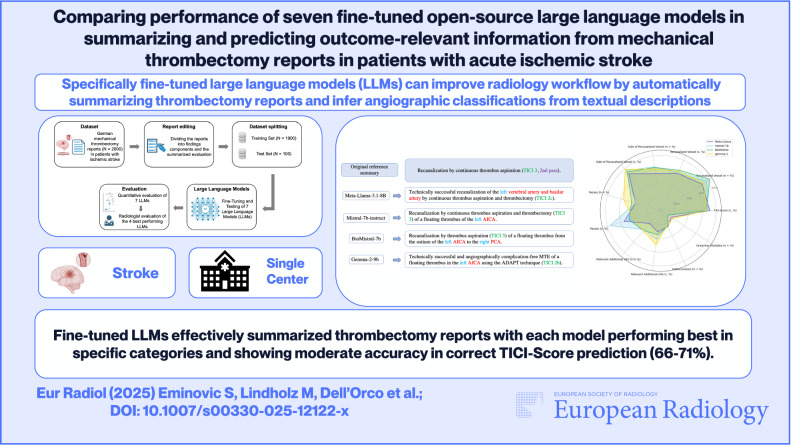

## Introduction

There is an increasing need for radiological examinations in line with population growth and demographic change [1]. This leads to an increasing volume of medical data presenting significant challenges for healthcare professionals in terms of efficient documentation, analysis, and communication. The increasing number of endovascular mechanical thrombectomies is in line with this trend [2–4], with a corresponding growing demand for neuro-interventionalists [5].

As a time-critical procedure for ischemic stroke, mechanical thrombectomy requires timely radiological reports to assess procedural outcomes and guide ongoing patient management. Post-mechanical thrombectomy reports are complex, containing multidimensional data that cover technical aspects, materials used, angiographic findings, imaging-based treatment outcome scores, interpretative insights, and recommendations for ongoing management, including follow-up care. Large Language Models (LLMs) have the capacity to process large text datasets, opening new possibilities for automating summarization and structured data extraction from free-text radiological reports [6–10], thereby enhancing the radiological workflow. Utilizing these models to generate structured outputs, such as ready-to-use report summaries, can support clinicians and improve overall healthcare efficiency. However, the use of LLM-generated summarization for thrombectomy reports has not yet been investigated. The increasing number of available LLMs presents heterogeneous capabilities, influenced by factors such as model architecture, training data and fine-tuning on medical datasets, all of which impact their suitability for radiological applications [11, 12]. Given the growing number of LLMs with varying capabilities, comparing these models is essential to identify the best-performing tool for summarizing mechanical thrombectomy reports. This study aims to systematically evaluate open-source LLMs for clinical use by assessing the correctness and completeness of their generated summaries for thrombectomy reports. The findings may provide insights for LLM integration into clinical practice, optimizing the time-critical communication of information in stroke care pathways by automating summarization for radiology reports. Furthermore, we hypothesize that LLMs can reliably predict the “Thrombolysis In Cerebral Ischemia” (TICI) score [13] from the report findings sections alone, despite this classification being based on the angiographic images that quantify the success of vessel reperfusion after the thrombectomy procedure. This allows us to specifically test the ability of LLMs to perform conceptual transfer and reasoning by inferring an angiographic classification from textual descriptions alone.

## Materials and methods

### Study design

This single-center retrospective study was approved by the ethics committee (Charité Berlin, Germany [protocol number EA1/115/24]), and written informed consent was waived by the institutional review boards.

### Data collection and analysis

**Fig. 1:**
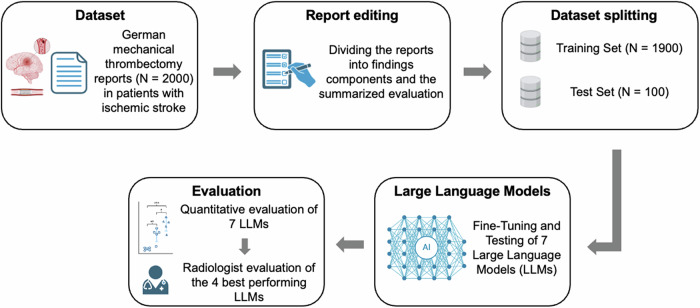
Illustration of the study process from data collection, processing/splitting, to fine-tuning and evaluation

A total of 2000 neurointerventional radiology reports for mechanical thrombectomy in patients with an acute ischemic stroke, written in German, were collected between September 2013 to August 2024 (study design is displayed in Fig. 1) and identified by their procedural code. Neurointerventional radiology reports for mechanical thrombectomy typically follow a standardized structure, consisting of the performing interventionalists, the technical details, the detailed findings section and the summarizing impression. This distinction ensures a clear separation between objective imaging findings and their clinical interpretation in the impression section. For analysis, the report was segmented into two components: (1) the findings sections of the respective case, including the performing interventionalists and the technical details, and (2) the summarizing impression. The impression should ideally describe whether the intervention was successful and whether it was performed with or without complications. It should describe the recanalized vessels and their side, specify the number of catheters passes required during intervention, and, if relevant, include additional information such as procedure-related complications. For training, each LLM received the findings as well as the impression sections. During testing, the models received only the findings section as input and generated the corresponding summary with the previously performed fine-tuning as the foundation.

### Training and testing

The dataset was split into a training set (*N* = 1900) for Low-Rank Adaptation (LoRA) [14] model fine-tuning and a test set (*N* = 100) for performance evaluation. All imaging reports were included via convenience sampling and randomly allocated between training and test cohorts to minimize selection bias and ensure comparable case distributions. Due to the limited transparency of closed-source models presenting significant challenges for implementation in medicine [15], we exclusively included open-source models. Seven open-source LLMs (five general and two medical domain) were selected for fine-tuning: Meta-Llama-3.1-8b-bnb-4bit [16, 17], mistral-7b-instruct-v0.3-bnb-4bit [18, 19], gemma-2-9b-bnb-4bit [20, 21], Llama-3.2-1B-bnb-4bit [22], Llama-3.2-3B-bnb-4bit [23], OpenBioLLM-8b [24] and BioMistral-7b [25] with OpenBioLLM-8b (based on Meta-Llama-3-8b models) and BioMistral-7b (based on Mistral-7b-Instruct-v0.1) already being fine-tuned for medical use-cases. These models were chosen for comparison due to their diverse architectures, varying sizes and specialization levels (general and medical domain). All models were fine-tuned to summarize the reports effectively while preserving clinical accuracy, as described in detail further below. Further details on the fine-tuning process are provided below.

#### Prompting

We instructionally prompted all seven LLMs via Application Programming Interface (API) in the exact same way, with (translated from German): *“Please summarize the neuroradiological intervention report findings in a clear, concise and medically accurate assessment. Pay particular attention to the TICI classification for the treatment success of a recanalization therapy, which is structured as follows: 0* = *No perfusion (0%) or anterograde flow distal to the occlusion site; 1 = Penetration but no perfusion. Contrast medium penetration exists distal to the obstruction but with minimal filling of the normal territory; 2a = Low perfusion with filling of distal branches of less than 50% of the visualized territory; 2b = Substantial perfusion with filling of distal branches of at least 50% of the visualized territory; 2c = Nearly complete perfusion with the exception of slow flow in a few distal cortical vessels or the presence of small distal cortical emboli; 3 = Complete perfusion with normal filling of all distal branches.”* We prompted them in the German language, focusing on summarizing the findings with particular emphasis on the TICI-Score. As the TICI classification has been modified twice (TICI from 2003 [13]), modified TICI-Score (mTICI from 2013 [26]) and expanded TICI (eTICI from 2019 [27]), we have explicitly explained every grade of the (e)TICI-Score in the prompt, so that the LLMs consider the grade 2c expansion of the (e)TICI-Score, which is used in the radiologists’ original summary.

#### Technical specificities

Within the training and evaluation process, all LLMs were initialized using Unsloth’s FastLanguageModel API [28], enabling efficient loading and processing with 4-bit quantization for memory efficiency and configured for a maximum sequence length of 4096 tokens. Quantization is a technique that reduces the precision of numerical representations, such as lowering floating-point precision from 16-bit or 32-bit to 4-bit or 8-bit to decrease memory usage and speed up computation [29]. We employed Parameter-Efficient Fine-Tuning (PEFT), which refers to the process of adjusting the parameters of a pre-trained large model to adapt it to a specific task or domain while minimizing the number of additional parameters introduced or computational resources required [30]. Specifically, we used LoRA, a fine-tuning method that keeps the original parameters frozen and adjusts only a relatively small number of extra parameters via matrix decomposition to optimize training on limited resources [14]. Models were fine-tuned using a learning rate of 3e-4, a batch size of 4, and mixed precision training (Brain Floating Point 16-bit or Half Precision Floating Point 16-Bit). All models were 4-bit quantized and fine-tuned with LoRA (rank r = 16, α = 16, dropout = 0) applied to every projection layer of each Transformer block (q_proj, k_proj, v_proj, up_proj, down_proj, o_proj, gate_proj) using the PEFT implementation in Unsloth with rslora and gradient checkpointing enabled. Tokenisation relied on the original SentencePiece tokenizer of each base model, wrapped with Unsloth’s ChatML template, and reproducibility was ensured by fixing random seeds at every level (NumPy = 1, dataset shuffle = 42, Trainer = 0). Training was conducted over three epochs using gradient accumulation for efficiency. The supplementary figure panel (Supplementary Fig. 1) illustrates each model’s training loss trajectory over three epochs. In all seven cases, the loss declines steadily and levels off without any significant rebounds, demonstrating stable convergence and no clear evidence of overfitting. Training was performed on four NVIDIA A100 Graphics Processor Units with optimized memory management via 4-bit quantization and gradient checkpointing. Our fine-tuning configuration file, the prompt template and evaluation script are available in GitHub (https://github.com//ibrarad/radiology-llm-finetuning-summarization).

### Study evaluation

A two-step evaluation process was used to assess the performance of all models in summarizing mechanical thrombectomy reports.

#### Quantitative evaluation

We evaluated the LLM-generated summaries of all seven LLMs with the original summary as a reference using the quantitative metrics Recall-Oriented Understudy for Gisting Evaluation Unigram-based (ROUGE-1), Recall-Oriented Understudy for Gisting Evaluation Bigram-based (ROUGE-2), Recall-Oriented Understudy for Gisting Evaluation Longest Common Subsequence (ROUGE-L) [31], Metric for Evaluation of Translation with Explicit Ordering (METEOR) [32], Bidirectional Encoder Representations from Transformers Score (BERTScore [F1]) [33] and BiLingual Evaluation Understudy (BLEU) [34]. These metrics collectively provide a robust framework to assess the quality of model-generated summaries in terms of content coverage, coherence, semantic similarity, and linguistic accuracy and are widely recognized for this purpose [35]. ROUGE-1, ROUGE-2, ROUGE-L [31] rely on exact word or phrase matches, focusing on lexical matching and not deeper semantics, with the following purpose: ROUGE-1 measures the overlap of unigrams (single words) between the model-generated summary and the reference (here: interventional neuroradiologist-generated) summary. It evaluates the ability to capture basic content. ROUGE-2 is similar to ROUGE-1, but it considers bigram (two-word) overlaps, providing insight into the preservation of short phrases and contextual accuracy. ROUGE-L measures the longest common subsequence between the generated and reference summaries. It assesses fluency and the logical coherence of the text [31]. METEOR, BERTScore (F1) und BLEU go beyond surface-level matching and evaluate the meaning or intent of the generated text: The METEOR score evaluates the quality of a summary based on unigram matches, incorporating synonyms, stemming, and paraphrasing, making it particularly suited for diverse linguistic variations [32]. BERTScore (F1) uses contextual embeddings from transformer models like BERT to compute the semantic similarity between the generated and reference summaries [33]. It focuses on meaning preservation and aligns with human judgment. The BLEU score evaluates the correspondence between the generated and reference summaries by analyzing n-gram precision [34].

Training and evaluation were performed with Python (version 3.10.14) using multiple packages: datasets (version 2.21.0), numpy [36](version 1.26.4), pandas [37] (version 2.2.3), pytorch [38] with pytorch-triton (version 3.0.0), torch (version 2.4.0), torchaudio (version 2.4.0), torchvision (version 0.19.0), transformers [39] (version 4.47.1,), trl [40] (version 0.10.1), unsloth [28] (2024.8) and evaluate (version 0.4.3).

#### Qualitative evaluation

The summaries (*N* = 100, from the test set) generated by the four best-performing LLMs (selected based on the aggregate score across all quantitative metrics, Supplementary Table S1) were further qualitatively evaluated by a second-year radiology resident (S.E.) and in cases of discrepancies between the LLM-generated information and the gold standard, an eighth-year diagnostic and interventional neuroradiologist (J.N.) was consulted for further evaluation. Comparing the four models with the highest quantitative scores in a further qualitative evaluation still provides a relatively large model sample, but despite their similar quantitative results, a thorough expert analysis can nonetheless reveal significant differences. In contrast to the quantitative evaluation (comparison of the generated summary vs. the original summarizing impression), the qualitative evaluation compared the generated summary with the detailed original findings. This evaluation focused on the completeness and correctness of key information included in the generated summary. The following aspects were assessed:

- TICI-score (correctness)
- Recanalized vessel (mentioned; if yes, correctness)
- Side of recanalized vessel (mentioned; if yes, correctness)
- Number of catheter passes during the intervention (mentioned; if yes, correctness)
- Relevant additional information (e.g., complications: problems in establishing an inguinal access route or causing a periinterventional dissection) (mentioned; if yes, completeness and/or correctness)
- Number of hallucinations
- Number of grammar mistakes

We carried out very detailed analyses for all categories (detailed overview table provided as Supplementary Table S2). For example, the TICI score was initially classified into: completely correct, partially correct, incorrect, not mentioned, or unclear (generated without reference).

**Fig. 2:**
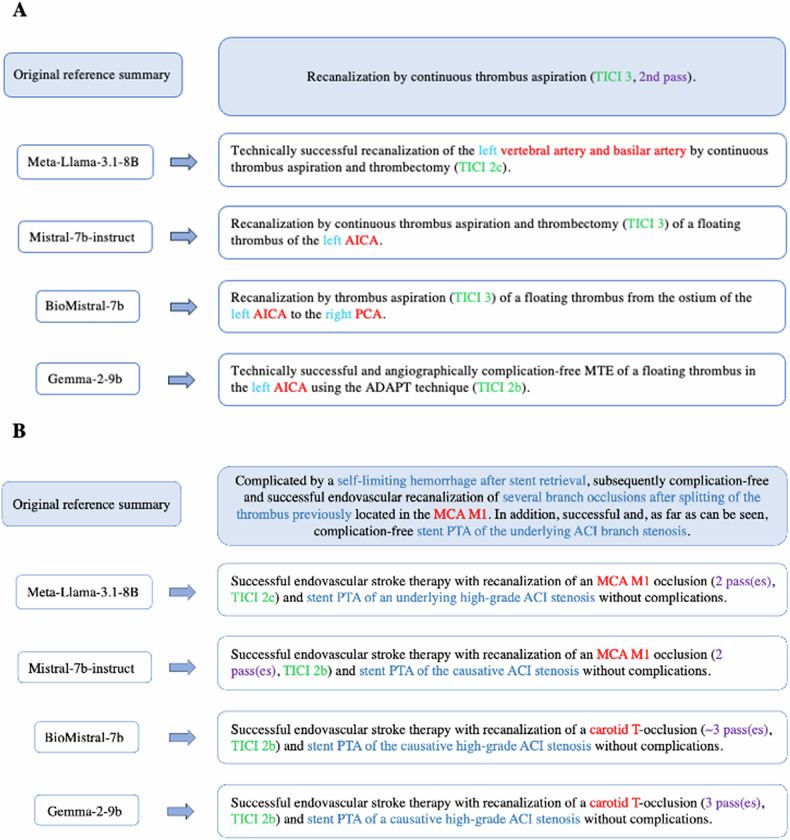
Display of exemplary original summaries with the specific generated summaries of each LLM (translated from German), with some of the manually analyzed categories marked in color (red: vessel, light blue: vessel side, green: TICI score, purple: number of passes, blue: additional information). **A** Compared to the relatively concise original summary, the LLMs have all described a recanalized vessel and the TICI-Score with slight differences. **B** No LLM mentioned the complication of the periinterventional, self-limiting hemorrhage and explicitly described that there was no complication. Furthermore, there are differences in the mentioned number of passes and the generated TICI score (TICI in this example without reference)

Of the *N* = 100 test set reports, *N* = 83 included a TICI score as a reference in the findings section, so only these were used in the radiologist evaluation in this category. Furthermore, important additional information was present only in *N* = 53 reports. Figure 2 provides examples of the generated summaries compared to the original summarizing impression section.

#### Statistical evaluation

For statistical testing, we binarized all categories into “completely correct” vs. “not completely correct,” as any incorrect or incomplete information poses a risk in critical interventional treatment. We applied a two-tailed, four-sample χ^2^ test to evaluate differences in the LLM’s performances. If the overall test indicated statistical significance (*p* < 0.05), a post hoc pairwise χ² test was conducted to determine whether differences between individual LLM pairs were statistically significant. We performed post hoc power analyses (two-sided McNemar test, α = 0.05, discordance = 25%) for representative binary outcomes with different test set sizes, demonstrating that power ranged from approximately 42 to 52% to detect 10 percentage-point differences and from 75 to 85% to detect 15 percentage-point differences between models. Accordingly, our study is adequately powered to reveal moderate performance gaps (≥ 15 pp), whereas smaller differences (around 10 pp) may be interpreted as exploratory. Analyses were performed and graphs and diagrams were created using Python (version 3.9.13) with multiple packages including Pandas (version 2.2.2), NumPy (version 1.20.0 and 1.23.1), SciPy (version 1.13.1) and Matplotlib (version 3.7.1 and 3.6.3). Figure 1 was created using items from biorender (https://app.biorender.com).

## Results

### Quantitative evaluation

**Fig. 3:**
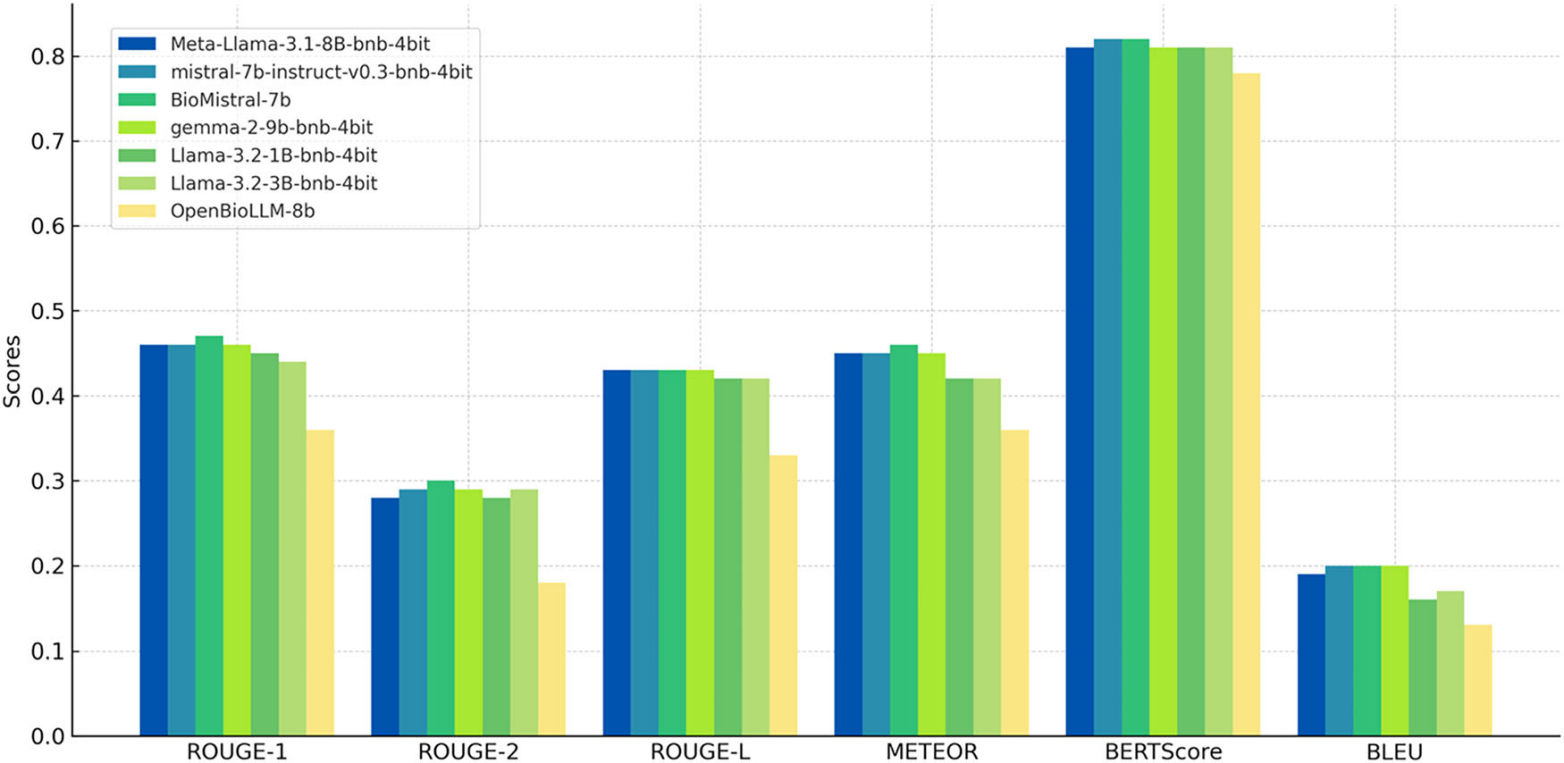
Bar charts displaying quantitative performance metrics for seven evaluated open-source LLMs (Meta-Llama-3.1-8B-bnb-4bit, mistral-7b-instruct-v0.3-bnb-4bit, BioMistral-7b, gemma-2-9b-bnb-4bit, Llama-3.2-1B-bnb-4bit, Llama-3.2-3B-bnb-4bit, OpenBioLLM-8b) in summarizing radiology reports for mechanical thrombectomy in patients with acute ischemic stroke

The performance of the seven LLMs that were evaluated using the quantitative metrics is detailed in Fig. 3 (numeric values are displayed in Supplementary Table S1). For ROUGE scores (ROUGE-1, ROUGE-2, ROUGE-L), all models performed similarly, with BioMistral-7b achieving the highest scores (ROUGE-1: 0.47, ROUGE-2: 0.30, ROUGE-L: 0.43). BioMistral-7b also led in METEOR, scoring 0.46. Most models achieved similar BERTScore (F1) values (0.81–0.82), with BioMistral-7b and mistral-7b-instruct demonstrating the best semantic alignment. BLEU scores were relatively low across models, with slight differences, and BioMistral-7b and mistral-7b-instruct again scoring the highest (BLEU: 0.20). OpenBioLLM-8B performed significantly worse than all other LLMs across every quantitative metric (Supplementary Table S2).

### Qualitative evaluation

**Fig. 4:**
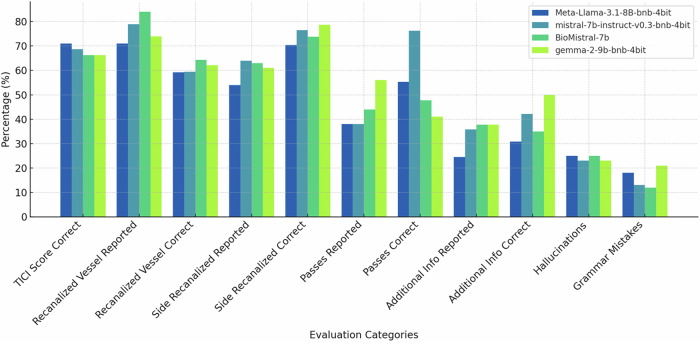
Bar charts displaying qualitative performance metrics (in %) for the manually evaluated results of the four best-performing LLMs in summarizing radiology reports for interventional acute ischemic stroke treatment

The four best-performing models, Meta-Llama-3.1-8b-bnb-4bit, mistral-7b-instruct-v0.3-bnb-4bit, gemma-2-9b-bnb-4bit, BioMistral-7b, were considered for further qualitative evaluation with all results being displayed in Table 1 (graphically displayed in Fig. 4). Statistically significant differences were found between the models in reporting the number of needed passes per intervention in the summary (*p* = 0.03) and in accuracy of those summaries (*p* < 0.01). Mistral-7b-instruct significantly more often described the number of passes correctly compared to gemma-2-9b (correct/reported [%]: 29/38 [76.32%] vs. 23/56 [41.07%], *p* < 0.01).

**Table 1:** Manual radiologists’ comparative analysis of completeness and correctness of the large language model-generated summaries

	Meta-Llama-3.1-8B-bnb-4bit	mistral-7b-instruct-v0.3-bnb-4bit	BioMistral-7b	gemma-2-9b-bnb-4bit	*p*-value χ^2^ test
TICI score, *n* (ref.: completely correct) (%)	*N* = 83	*N* = 83	*N* = 83	*N* = 83	
Yes	59 (71.08%)	57 (68.67%)	55 (66.27%)	55 (66.27%)	0.89 (n.s.)
No	24 (28.92%)	26 (31.33%)	28 (33.73%)	28 (33.73%)	
Recanalized vessel, *n* (ref.: reported in LLM-generated summary)	*N* = 100	*N* = 100	*N* = 100	*N* = 100	
Yes	71 (71%)	79 (79%)	84 (84%)	74 (74%)	0.14 (n.s.)
No	29 (29%)	21 (21%)	16 (16%)	26 (26%)	
Recanalized vessel, *n* (ref.: completely correct)	*N* = 71	*N* = 79	*N* = 84	*N* = 74	
Yes	42 (59.15%)	47 (59.49%)	54 (64.29%)	46 (62.16%)	0.90 (n.s.)
No	29 (40.85%)	32 (40.51%)	30 (35.71%)	28 (37.84%)	
Side of recanalized vessel, *n* (ref.: reported in LLM-generated summary)	*N* = 100	*N* = 100	*N* = 100	*N* = 100	
Yes	54 (54%)	64 (64%)	63 (63%)	61 (61%)	0.47 (n.s.)
No	46 (46%)	36 (36%)	37 (37%)	39 (39%)	
Side of recanalized vessel, *n* (ref.: completely correct)	*N* = 54	*N* = 64	*N* = 65	*N* = 61	
Yes	38 (70.37%)	49 (76.56%)	48 (73.85%)	48 (78.69%)	0.76 (n.s.)
No	16 (29.63%)	15 (23.44%)	17 (26.15%)	13 (21.31%)	
Passes, *n* (ref.: reported in LLM-generated summary)	*N* = 100	*N* = 100	*N* = 100	*N* = 100	
Yes	38 (38%)	38 (38%)	44 (44%)	56 (56%)	0.03*
No	62 (62%)	62 (62%)	56 (56%)	44 (44%)	
Passes, *n* (ref.: completely correct)	*N* = 38	*N* = 38	*N* = 44	*N* = 56	
Yes	21 (55.26%)	29 (76.32%)	21 (47.73%)	23 (41.07%)	< 0.01**
No	17 (44.74%)	9 (23.68%)	23 (52.27%)	33 (58.93%)	
Relevant additional information (ref.: reported in LLM-generated summary)	*N* = 53	*N* = 53	*N* = 53	*N* = 53	
Yes	13 (24.53%)	19 (35.85%)	20 (37.74%)	20 (37.74%)	0.41 (n.s.)
No	40 (75.47%)	34 (64.15%)	33 (62.26%)	33 (62.26%)	
Relevant additional information, *n* (ref.: completely correct)	*N* = 13	*N* = 19	*N* = 20	*N* = 20	
Yes	4 (30.77%)	8 (42.11%)	7 (35%)	10 (50%)	0.68 (n.s.)
No	9 (69.23%)	11 (57.89%)	13 (65%)	10 (50%)	
Hallucinations, *n*	*N* = 100	*N* = 100	*N* = 100	*N* = 100	
Yes	25 (25%)	23 (23%)	25 (25%)	23 (23%)	0.97 (n.s.)
No	75 (75%)	77 (77%)	75 (75%)	77 (77%)	
Grammar mistakes, *n*	*N* = 100	*N* = 100	*N* = 100	*N* = 100	
Yes	18 (18%)	13 (13%)	12 (12%)	21 (21%)	0.26 (n.s.)
No	82 (82%)	87 (87%)	88 (88%)	79 (79%)	

χ^2^: chi-square

*: significant (*p* < 0.05); **: *p* < 0.01; n.s.: *p* > 0.05

Bold *p* values indicate statistically significant results

In none of the other categories was a significant difference observed. Correct TICI scores ranged from 66.27 to 71.08% across models, with Meta-Llama-3.1 performing the best by correctly deriving the score in 71.08% (*p* = 0.89). The recanalized vessels were reported within a range from 71 to 84% (*p* = 0.14) with BioMistral-7b achieving the highest percentage (84%). Of those reported between 59.15 and 64.29% were correctly summarized with BioMistral-7b again achieving the highest percentage (*p* = 0.90). For the anatomical side, the reporting rates ranged from 54 to 64% with mistral-7b-instruct achieving the highest rate (64%; *p* = 0.47). Reporting the correct recanalization side ranged from 70.37 to 78.69% with gemma-2-9b performing best (*p* = 0.76, Table 2). Relevant additional information was infrequently reported in the summary, ranging from 24.53 to 37.74%, with BioMistral-7b and gemma-2-9b tied at 37.74% (*p* = 0.41). Gemma-2-9b had the highest rate of completely correct mentions at 50% (*p* = 0.68). Hallucination rates were moderate and consistently found across all models (23–25%; *p* = 0.97). We systematically categorized hallucinations and summarized their relative frequencies in Table 3. The most prevalent error types were incorrect recanalized vessel side (44.74% of all hallucinations) and hallucinated recanalized vessels including vessel segments (30.70%). Notable hallucinations included Passes (4.39%), Groin‑to‑Perfusion timing (2.63%), as well as hallucinated extent of occlusions (2.63%). Multi-label errors, where a single summary contained two simultaneous hallucinations, occurred in 6.14%.

**Table 2:** Display of *p*-values of post hoc pairwise χ^2^ test for those summary categories which statistically significant differences

*p*-values post hoc pairwise χ^2^ test with Holm correction				
	Meta-Llama-3.1-8B-bnb-4bit	mistral-7b-instruct-v0.3-bnb-4bit	BioMistral-7b	gemma-2-9b-bnb-4bit
Number of mentioned passes				
Meta-Llama-3.1-8B-bnb-4bit	-	1.00 (n.s.)	1.00 (n.s.)	0.10 (n.s)
mistral-7b-instruct-v0.3-bnb-4bit	-	-	1.00 (n.s.)	0.10 (n.s)
BioMistral-7b	-	-	-	0.48 (n.s.)
gemma-2-9b-bnb-4bit	-	-	-	-
Correctness of mentioned passes				
Meta-Llama-3.1-8B-bnb-4bit	-	0.36 (n.s.)	1.00 (n.s.)	0.76 (n.s.)
mistral-7b-instruct-v0.3-bnb-4bit	-	-	0.07 (n.s.)	< 0.01**
BioMistral-7b	-	-	-	1.00 (ns)
gemma-2-9b-bnb-4bit	-	-	-	-

χ^2^: chi-square

*: significant (*p* < 0.05); **: *p* < 0.01; n.s.: *p* > 0.05

Bold *p* values indicate statistically significant results

**Table 3:** Frequency and relative proportion of hallucination types across qualitatively evaluated LLMs

Hallucination type	Meta-Llama	mistral-7b	BioMistral	Gemma-2	Overall, *n*
Recanalized vessel, *n* (%)	11 (31.43%)	9 (25.71%)	8 (22.86%)	7 (20.00%)	35 (30.70%)
Side, *n* (%)	12 (23.53%)	13 (25.49%)	13 (25.49%)	13 (25.49%)	51 (44.74%)
Passes, *n* (%)	1 (20.00%)	1 (20.00%)	1 (20.00%)	2 (40.00%)	5 (4.39%)
Groin-to-perfusion, *n* (%)	1 (33.33%)	0 (0.00%)	2 (66.67%)	0 (0.00%)	3 (2.63%)
Extent of occlusion, *n* (%)	0 (0.00%)	1 (33.33%)	0 (0.00%)	2 (66.67%)	3 (2.63%)
Therapy course, *n* (%)	0 (0.00%)	0 (0.00%)	3 (100.00%)	0 (0.00%)	3 (2.63%)
Multiple hallucinations per summary, *n* (%)	1 (14.29%)	1 (14.29%)	3 (42.86%)	2 (28.57%)	7 (6.14%)
• Vessel + side (%)	1 (16.67)	1 (16.67)	1 (16.67)	2 (33.33%)	6 (5.26%)
• Passes + therapy course (%)	0 (0.00%)	0 (0.00%)	1 (100.00%)	0 (0.00%)	1 (0.88%)

Grammar mistake rates ranged from 12 to 21% with gemma-2-9b having the highest rate (21%; *p* = 0.26).

## Discussion

This study evaluated the performance of seven open-source, fine-tuned LLMs in summarizing and predicting outcome-relevant information from neurointerventional radiology reports for mechanical thrombectomy in patients with an acute ischemic stroke.

Our primary objective was to systematically compare the performance of different LLMs in summarizing specialized medical information after being fine-tuned for this use-case, with the four best-performing models undergoing manual evaluation. As a secondary objective, we analyzed these top four LLMs for their ability to predict the TICI score, an angiography-based outcome measure of post-thrombectomy reperfusion, using only the report findings to assess conceptual transferring and reasoning.

All seven open-source models showed the ability to summarize mechanical thrombectomy reports with relatively similar results in quantitative metrics, with OpenBioLLM-8b performing moderately worse. Within the four best-performing models, Meta-Llama-3.1-8b-bnb-4bit, mistral-7b-instruct-v0.3-bnb-4bit, gemma-2-9b-bnb-4bit and BioMistral-7b, most categories did not show statistically significant differences, suggesting similar performance across the LLMs, with every LLM achieving the best result in at least one category. This suggests that fine-tuning pre-trained LLMs on specialized medical datasets can lead to a convergence in performance, enabling them to achieve similar results across various tasks. Studies show that medical-domain fine-tuning can align model performance between LLMs and also enables smaller fine-tuned models to outperform larger models without fine-tuning [41, 42]. The only significant differences were that Gemma-2-9b most often reported the number of passes, while Mistral-7B-Instruct was most accurate about it. All four LLMs performed moderately well in predicting the TICI score from the findings section alone, ranging from 66 to 71% correctness rate, underscoring the potential of LLMs to classify recanalization success purely from textual data.

Most existing literature on radiological summarization focuses on simplifying language for better patient understanding [43–46], less often for medical experts to improve the workflow [6–9], and especially not for neurointerventional radiology as in this study.

**Fig. 5:**
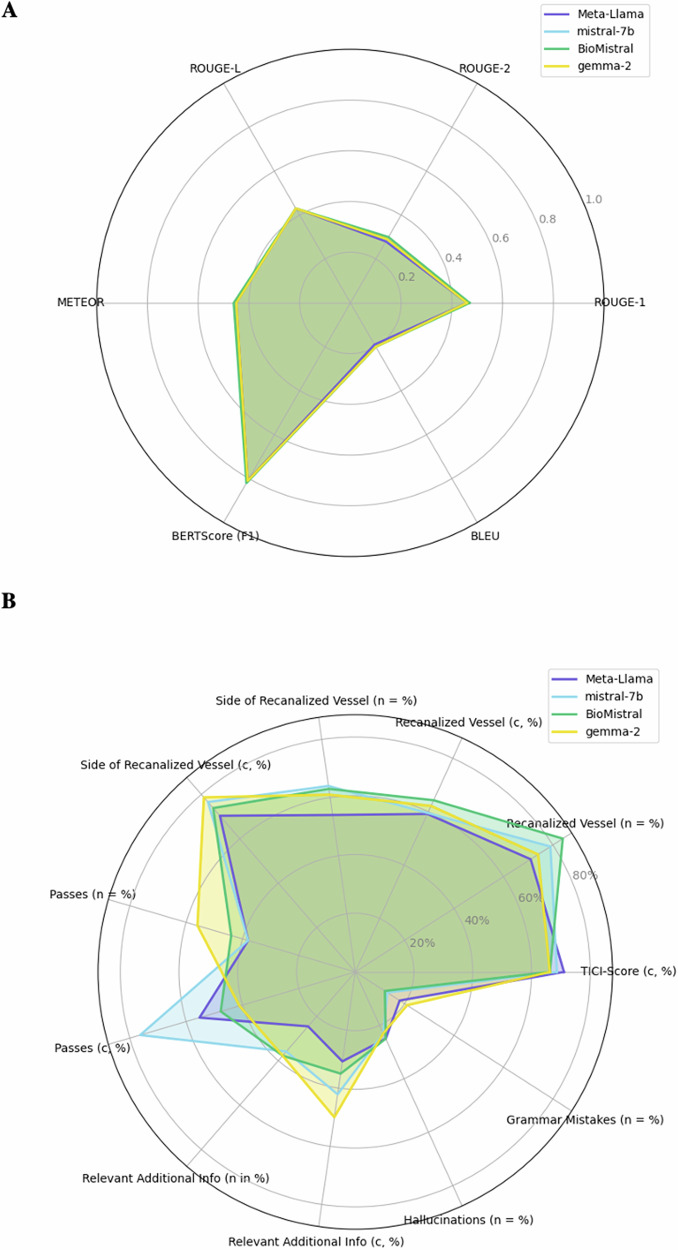
Radar diagram that displays **A** quantitative metrics and **B** manually evaluated results of the four best-performing LLMs in summarizing radiology reports for interventional acute ischemic stroke treatment. **A** shows very similar performance with very nuanced differences (Meta-Llama-3.1-8b-bnb-4bit, mistral-7b-instruct-v0.3-bnb-4bit, gemma-2-9b-bnb-4bit, BioMistral-7b). **B** displays greater deviations in the evaluated categories, especially in comparison to **A**. The information is given in %, a distinction is made between the proportion of reported events (n; *n* = % means that the number of reported events also corresponds to the % figure because we tested on 100 reports (= 100%), i.e., Meta-Llama-3.1-8b mentioned the vessel in 71 cases out of 100 reports which also corresponds to 71% of the cases). (c, %) indicates how many of the reported events were correctly reported (c is the abbreviation for correctness)

Quantitative metrics were very similar across the four best-performing LLMs (Fig. 5A). However, the manual evaluation displayed clear, albeit only a few significant differences (Fig. 5B). While quantitative metrics provide a standardized comparison, this highlights the question of whether they can adequately reflect the heterogeneity of complex medical data. It should be noted that for the quantitative metrics, the original summarizing impression section was used as a reference, and for the manual evaluation, the original findings, so comparability may be limited. Still, the discrepancy highlights the importance of qualitative analyses for complex medical texts. The concern of poor correlation with human judgment is also reflected in the literature, which describes a growing demand for automated assessment methods that can increasingly match human assessments, but are more efficient and cost-effective [47–50].

The ability of LLMs to identify and prioritize relevant additional findings as procedure-related complications was highly inconsistent. In many instances, only one model accurately described a relevant supplementary finding, reflecting difficulties in assigning significance to diverse medical data, likely due to the complexity and variability. This aligns with research suggesting LLMs can lack contextual understanding and perform poorly in higher-order reasoning [51, 52]. Conversely, when a structured, well-defined metric like the TICI score was explicitly mentioned in the report, all four LLMs incorporated this information into their summaries, demonstrating reliable processing of unambiguous data; however, there is a need for improved prioritization and contextual understanding in LLM training [51, 53], particularly when processing heterogeneous data. Despite those challenges and considering that TICI-scoring is highly interventionalist-dependent and based on the angiographic images, the LLMs still performed reasonably well in predicting the correct TICI score from the findings section alone. However, inferring TICI from free-text reports alone may introduce variability due to heterogeneous reporting practices or incomplete descriptions, which could affect clinical decision-making. Future work should therefore explore multimodal approaches that combine automatic text analysis with direct image evaluation to improve the robustness and clinical validity of TICI-score determination.

LLMs can generate factually incorrect outputs called hallucinations [54, 55], which is especially concerning in medicine, when clinical decision-making relies on precise data. Our observed hallucination rates of 23–25% across all four models lie within the broad range reported for medical text summarization [56, 57], with hallucinated vessel side (44.74%) and recanalized vessel (30.70%) accounting for the majority of all observed hallucinations. These errors likely reflect the frequent emphasis and repetition of laterality and vessel terminology in radiology reports, and especially in the case of recanalized vessels, the large variety of possible segment names (e.g., M1, A1, P2), which increases the model’s tendency to combine terms incorrectly. Example hallucinations include mistral-7b-instruct incorrectly describing the Arteria pericallosa and A1-segment without those ever being mentioned in the report findings, incorrect details on the “time from groin-to-perfusion” (hallucinated twice by BioMistral-7b) and also incorrect description of an intervention outcome (successful classified as unsuccessful). Rigorous validation and regular monitoring may help prevent the propagation of hallucinations in clinical settings. Concrete strategies for avoiding hallucinations for report summarizing settings may include the following: assigning model-derived confidence scores to each generated statement and flagging or suppressing outputs that fall below a predefined threshold (Confidence Scoring); restricting the model’s outputs to a curated repository of validated anatomical and procedural facts to prevent invention of nonexistent structures or outcomes (Knowledge Constraints); optimizing prompts with clear instructions and illustrative examples to ground summaries in source data and discourage speculative language (Refined Prompt Engineering); Incorporating retrieval-augmented generation to include the most relevant report passages in the prompt, ensuring that critical details such as vessel names and intervention outcomes are sourced directly from the original text; fine-tuning the model on a small, expert-annotated dataset where correct versus false summaries are explicitly labeled, thereby teaching the model to distinguish factual information from potential hallucinations.

Since the number of passes was not always explicitly reported, the models frequently diverged in their interpretations, with gemma-2-9b trying significantly more often to derive the exact number of passes. This variability reflects the difficulty of deriving implicit information by inferring it from context [51, 53].

Most summaries were grammatically correct (mostly simple mistakes, e.g., missing punctuation marks). However, there was a very lengthy, complex report (without exceeding maximum input sequence length) with the generated summaries truncating mid-sentence. This occurred with both mistral-7b and BioMistral, indicating that complex cases may exceed the processing capacity of certain LLMs or their ability to maintain coherence over extended text.

In a routine clinical setting, the LLM can be embedded in the RIS/PACS to auto-generate draft impressions immediately after image acquisition. Beyond generating report summaries, the system can extract structured parameters (occlusion site, TICI grade, pass count, laterality) in machine-readable form, enabling downstream applications such as clinical research data pipelines or automated registry and quality-assurance reporting. In these use-cases, a radiologist should perform the final validation and sign-off of all generated outputs.

LLM-generated errors in report impressions can mislead downstream care and follow-up-decision such as unnecessary imaging or wrong-site interventions. Extending the use-case to structured data extraction, incorrect extracted parameters may propagate into research databases, automated registries, and quality-assurance metrics, undermining study validity and performance monitoring; therefore, radiologist review remains essential.

A moderate rate of LLM-generated hallucinations increases the time required to review each case due to the need for more comprehensive checks. By implementing a confidence-scoring mechanism that flags only low-certainty statements, radiologists can concentrate their attention on the most unreliable outputs, minimizing additional review time and preserving a net efficiency gain.

## Limitations

This study has several limitations. This study is constrained by its single-center design and relatively modest sample size, which may reflect homogeneous reporting styles given the limited number of contributing interventionalists. However, Charité is among the busiest neurointerventional stroke centers, performing a substantial annual volume of mechanical thrombectomies with a varied team of specialists. This combination of high procedural throughput and practitioner diversity helps to partially offset the homogeneity inherent in single-site datasets. To simplify interpretability, we categorized model outputs only in two categories as “completely correct” or “not completely correct” with the latter including partially correct, not mentioned and ultimately unclear information. While this approach ensured stricter standards, it may have underestimated model performance by failing to distinguish minor inaccuracies from significant errors. While PEFT reduces memory, computation needs and improves adaptability, it may not always achieve the same performance as full fine-tuning.

Furthermore, the lack of a separate validation cohort prevented continuous overfitting assessment, and although our 100-report test set was randomly sampled to reflect the overall distribution, its relatively small size may limit the generalizability of our findings. The models faced limitations due to their size and 4-bit quantization, which, while computationally efficient, can hinder context retention and the accurate capture of medical details. Compared to larger architectures, this may reduce representational accuracy and increase errors in complex medical texts. The original summarizing impressions varied and were partially incomplete—as these were used for training and for quantitative evaluation, this could introduce biases and lead to inconsistent model performance. Furthermore, our prompt was very specific on the TICI score but lacked an example summary, which could have led to more consistency in the summaries generated.

## Conclusion

Our findings illustrate the potential of use-case-specific fine-tuned LLMs to enhance the radiology workflow by generating radiology report summaries while simultaneously highlighting the limitations in summarizing complex medical datasets, particularly when incorporating multimodal data such as the TICI score. While structured and unambiguous information is reliably incorporated, challenges remain in handling heterogeneity, prioritizing additional findings, and avoiding hallucinations. These insights underscore the need for further refinement of LLMs, with a focus on contextual understanding, robustness in handling ambiguous inputs, and safeguards against hallucinated information. Future research should prioritize the development of domain-specific models and datasets to address these challenges and improve the reliability of LLMs in clinical practice.

## Supplementary information

ELECTRONIC SUPPLEMENTARY MATERIAL

## Abbreviations

API: Application programming interface
BERTScore: Bidirectional Encoder Representations from Transformers Score
BLEU: Bilingual evaluation understudy
eTICI: Expanded thrombolysis in cerebral ischemia
LLM: Large language model
LoRA: Low-rank adaptation
METEOR: Metric for evaluation of translation with explicit ordering
mTICI: Modified thrombolysis in cerebral ischemia
PEFT: Parameter-efficient fine-tuning
ROUGE-1: Recall-oriented understudy for gisting evaluation (unigram-based)
ROUGE-2: Recall-oriented understudy for gisting evaluation (bigram-based)
ROUGE-L: Recall-oriented understudy for gisting evaluation (longest common subsequence)
TICI: Thrombolysis in cerebral ischemia
