# Supplementary material for: Comparing performance of seven fine-tuned open-source large language models in summarizing and predicting outcome-relevant information from mechanical thrombectomy reports in patients with acute ischemic stroke
Source: Eur Radiol. 2025 Nov 17;36(5):3661–75. doi: 10.1007/s00330-025-12122-x (PMC13086895; doi:10.1007/s00330-025-12122-x)
Supplement: Supplementary file 1 — ELECTRONIC SUPPLEMENTARY MATERIAL [file 330_2025_12122_MOESM1_ESM.pdf]

Comparing Performance of Seven Fine-Tuned Open-Source Large Language Models in Summarizing and Predicting Outcome-Relevant Information from Mechanical Thrombectomy Reports in Patients with Acute Ischemic Stroke

ELECTRONIC SUPPLEMENTARY MATERIAL

Eur Radiol (2025) Eminovic S, Lindholz M, Dell‘Orco A, et al.

Supplementary Table S1 Quantitative evaluation of all Large language model-generated summaries

Modelle	ROUGE-1	ROUGE-2	ROUGE-L	METEOR	BERTScore (F1)	BLEU
Meta-Llama-3.1-8B-bnb-4bit	0.46	0.28	0.43	0.45	0.81	0.19
mistral-7b-instruct-v0.3-bnb-4bit	0.46	0.29	0.43	0.45	0.82	0.20
gemma-2-9b-bnb-4bit	0.46	0.29	0.43	0.45	0.81	0.20
Llama-3.2-1B-bnb-4bit	0.45	0.28	0.42	0.42	0.81	0.16
Llama-3.2-3B-bnb-4bit	0.44	0.29	0.42	0.42	0.81	0.17
OpenBioLLM-8b	0.36	0.18	0.33	0.36	0.78	0.13
BioMistral-7b	0.47	0.30	0.43	0.46	0.82	0.20

Supplementary Table S2 P values Friedman and pairwise Wilcoxon Signed-Rank test with Holm correction for Quantitative Evaluation Metrics

**P values Friedman and pairwise Wilcoxon Signed-Rank test with Holm correction for Quantitative Evaluation Metrics**	
**ROUGE-1, -2, -L: Friedman test p < 0.01****	
Wilcoxon Signed-Rank test with Holm correction	
	OpenBioLLM-8B
BioMistral-7B	< 0.01 **
Llama-3.2-1B-bnb-4bit	< 0.01 **
Llama-3.2-3B-bnb-4bit	< 0.01 **
Meta-Llama-3.1-8B-bnb-4bit	< 0.01 **
gemma-2-9b-bnb-4bit	< 0.01 **
mistral-7b-instruct-v0.3-bnb-4bit	< 0.01 **
**METEOR: Friedman test p < 0.01****	
Wilcoxon Signed-Rank test with Holm correction	
	OpenBioLLM-8B
BioMistral-7B	< 0.01 **
Llama-3.2-1B-bnb-4bit	< 0.01 **
Llama-3.2-3B-bnb-4bit	0.04 *
Meta-Llama-3.1-8B-bnb-4bit	< 0.01 **
gemma-2-9b-bnb-4bit	< 0.01 **
mistral-7b-instruct-v0.3-bnb-4bit	< 0.01 **
**METEOR: Friedman test p < 0.01****	
Wilcoxon Signed-Rank test with Holm correction	
	OpenBioLLM-8B
BioMistral-7B	< 0.01 **
Llama-3.2-1B-bnb-4bit	< 0.01 **
Llama-3.2-3B-bnb-4bit	< 0.01 **
Meta-Llama-3.1-8B-bnb-4bit	< 0.01 **
gemma-2-9b-bnb-4bit	< 0.01 **
mistral-7b-instruct-v0.3-bnb-4bit	< 0.01 **

*: significant (p < 0.05); **: p < 0.01

Supplementary Table S3 Detailed, comparative overview of completeness and correctness of the Large language model-generated summaries

	**Meta-Llama-3.1-8B-bnb-4bit**	**mistral-7b-instruct-v0.3-bnb-4bit [%]**	**BioMistral-7b [%]**	**gemma-2-9b-bnb-4bit [%]**
TICI-Score, n (ref.: correct)	N = 83	N = 83	N = 83	N = 83
yes (completely)	59 (71.08%)	57 (68.67%)	55 (66.27%)	56 (66.27%)
partially	3 (3.61%)	5 (6.02%)	4 (4.82%)	4 (4.82%)
incorrect	16 (19.28%)	13 (15.66%)	15 (18.07%)	17 (20.48%)
not mentioned	5 (6.02%)	8 (9.64%)	9 (10.84%)	7 (8.43%)
Recanalized vessel, n (ref.: reported in LLM-generated summary)	N = 100	N = 100	N = 100	N= 100
yes	71 (71.00%)	79 (79.00%)	84 (84.00%)	74 (74.00%)
no	29 (29.00%)	21 (21.00%)	16 (15.00%)	26 (26.00%)
Recanalized vessel, n (ref.: completely correct)	N = 71	N = 79	N = 84	N = 74
yes (completely)	42 (59.15%)	47 (59.49%)	54 (64.29%)	46 (62.16%)
partially	25 (35.21%)	28 (35.44%)	23 (27.38%)	20 (27.03%)
incorrect	3 (4.23%)	3 (3.80%)	6 (7.14%)	6 (8.11%)
unclear	1 (1.41%)	1 (1.27%)	1 (1.19%)	2 (2.70%)
Side of recanalized vessel, n (ref.: reported in LLM-generated summary)	N = 100	N = 100	N = 100	N = 100
yes	47 (47.00%)	56 (56.00%)	59 (59.00%)	57 (57.00%)
Unilateral (A. basilaris)	7 (7.00%)	8 (8.00%)	4 (4.00%)	4 (4.00%)
partially	0 (0.00%)	0 (0.00%)	2 (2.00%)	0 (0.00%)
no	46 (46.00%)	36 (36.00%)	35 (35.00%)	39 (39.00%)
Side of recanalized vessel, n (ref.: completely correct)	N = 54	N = 64	N = 65	N = 61
yes (completely)	38 (70.37%)	49 (76.56%)	48 (73.85%)	48 (78.69%)
partially	4 (7.41%)	2 (3.13%)	3 (4.62%)	1 (1.64%)
incorrect	5 (9.26%)	3 (4.69%)	5 (7.69%)	3 (4.92%)
unclear	7 (12.96%)	10 (15.63%)	9 (13.85%)	9 (14.75%)
Passes, n (ref.: reported in LLM-generated summary)	N = 100	N = 100	N = 100	N = 100
yes	38 (38.00%)	38 (38.00%)	44 (44.00%)	56 (56.00%)
no	62 (62.00%)	62 (62.00%)	56 (56.00%)	44 (44.00%)
Passes, n (ref.: correct of reported)	N = 38	N = 38	N = 44	N = 56
yes (completely)	21 (55.26%)	29 (76.32%)	21 (47.73%)	23 (41.07%)
incorrect	5 (13.16%)	3 (7.89%)	5 (11.36%)	5 (8.93%)
Partially	0 (0.00%)	1 (2.63%)	1 (2.27%)	2 (3.57%)
unclear	12 (31.58%)	5 (13.16%)	17 (38.64%)	26 (46.43%)
Relevant additional information (ref.:	N = 53	N = 53	N = 53	N = 53

reported in LLM-generated summary)				
yes	13 (24.53%)	19 (35.85%)	20 (37.74%)	20 (37.74%)
no	40 (75.47%)	34 (64.15%)	33 (62.26%)	33 (62.26%)
Relevant additional information, n (ref.: correct of reported)	N = 13	N = 19	N = 20	N = 20
yes (completely)	4 (30.77%)	8 (42.11%)	7 (35.00%)	9 (45.00%)
incorrect	0 (0.00%)	1 (5.26%)	0 (0.00%)	0 (0.00%)
partially	9 (69.23%)	10 (52.63%)	13 (65.00%)	10 (50.00%)
extended	0 (0.00%)	0 (0.00%)	0 (0.00%)	1 (5.00%)
Hallucinations, n	N = 100	N = 100	N = 100	N = 100
yes	25 (25.00%)	23 (23.00%)	25 (25.00%)	23 (23.00%)
no	75 (75.00%)	77 (77.00%)	75 (75.00%)	77 (77.00%)
Grammar mistakes, n	N = 100	N = 100	N = 100	N = 100
yes	18 (18.00%)	13 (13.00%)	12 (12.00%)	21 (21.00%)
no	82 (82.00%)	87 (87.00%)	88 (88.00%)	79 (79.00%)

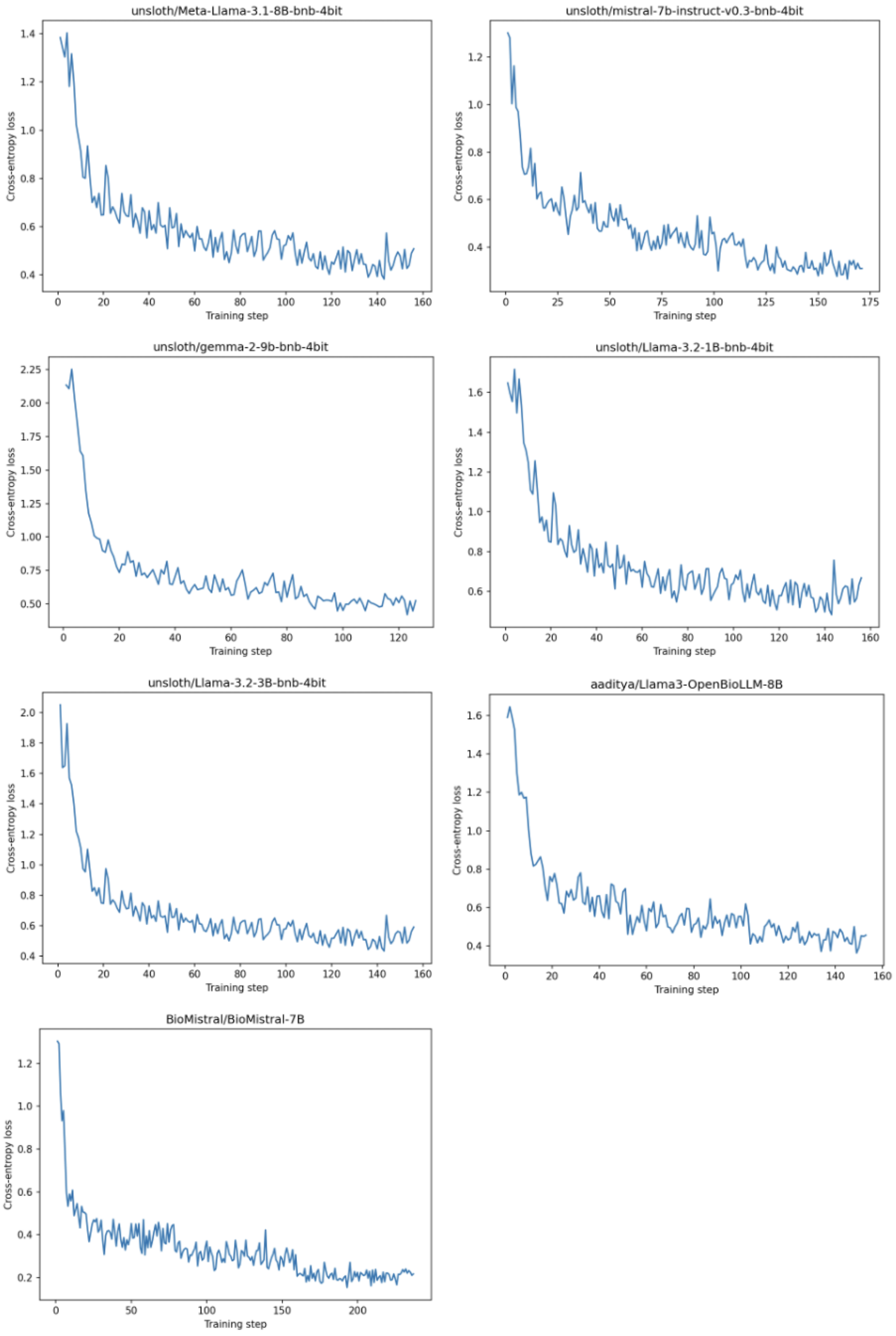

Supplementary Figure S1. Training loss curves for all seven fine-tuned LLMs over three epochs. Each subplot shows the per-step loss (y-axis) as a function of training step (x-axis) for Meta-Llama-3.1-8B-bnb-4bit, mistral-7b-instruct-v0.3-bnb-4bit, BioMistral-7b, gemma-2-9b-bnb-4bit, Llama-3.2-1B-bnb-4bit, Llama-3.2-3B-bnb-4bit, and OpenBioLLM-8b. In every case the loss decreases rapidly during early training and then plateaus smoothly without notable spikes or rebounds, indicating stable convergence and no signs of overfitting.

Eur Radiol (2025) Eminovic S, Lindholz M, Dell‘Orco A, et al.
